# In vivo transit dosimetry methodology for whole breast intensity modulated radiation therapy

**DOI:** 10.1002/acm2.70072

**Published:** 2025-03-26

**Authors:** Lucia Zirone, Elisa Bonanno, Giuseppina R. Borzì, Nina Cavalli, Alessia D'Anna, Andrea Girlando, Martina Pace, Giuseppe Stella, Carmelo Marino

**Affiliations:** ^1^ Medical Physics Department Humanitas Istituto Clinico Catanese Misterbianco Italy; ^2^ University of Catania Department of Physics and Astronomy “Ettore Majorana” Catania Italy; ^3^ Radiotherapy Department Humanitas Istituto Clinico Catanese Misterbianco Italy

**Keywords:** EPID, gamma passing rate, in vivo transit dosimetry

## Abstract

**Background:**

In vivo transit dosimetry using an electronic portal imaging device (EPID‐IVTD) is an important tool for verifying the accuracy of radiation therapy treatments. Despite its potential, the implementation of EPID‐IVTD in breast intensity modulated radiation therapy (IMRT) treatments has not yet been standardized, limiting its clinical adoption. A standardized EPID‐IVTD method could enhance treatment accuracy and improve patient safety in routine clinical practice.

**Purpose:**

This study aims to develop a method for EPID‐IVTD for whole breast IMRT treatment.

**Methods:**

Gamma passing rates (GPRs) analysis was the basis of the work conducted on a dataset of 50 patients. The first phase of the work focused on the identification of the reference fraction. In the second phase a method for performing EPID‐IVTD was implemented. Lower‐tolerance and ‐action limits (l‐TL and l‐AL), as introduced by AAPM TG 218, were employed to determine the reference fraction and used as alert and alarm thresholds, respectively, in EPID‐IVTD monitoring.

**Results:**

The first treatment fraction demonstrated the best dosimetric agreement with the theoretical plan and was therefore used as the reference in the second phase of the study. EPID‐IVTD results showed that 75% of the GPRs ranged from 97.5% to 99.9%, 93.83% were above the l‐TL, 4.31% fell between l‐TL and l‐AL, and 1.86% were below l‐AL.

**Conclusions:**

A method for the implementation of an effective EPID‐IVTD in whole breast IMRT treatment was developed and is now routinely applied at our center, enabling efficient monitoring in clinical practice.

## 1 INTRODUCTION

In vivo dosimetry (IVD) and in vivo transit dosimetry (IVTD) are essential practices in radiation therapy aimed at ensuring the accuracy and safety of treatments. The primary objectives of IVD and IVTD are to verify that the treatment complies with the established plan, identify and promptly correct any discrepancies, and optimize the efficacy of radiotherapy.^1^, ^2^, ^3^

In all areas of radiotherapy—whether intraoperative radiotherapy, intraoperative electron radiotherapy, brachytherapy, or external beam radiotherapy—the main goal of IVD is to monitor the actual radiation dose delivered to the patient during treatment to ensure that it conforms to the prescribed dose. To achieve this, various dosimeters are placed on the surface or inside the patient's body, including thermoluminescent dosimeters (TLDs), optically stimulated luminescence dosimeters (OSLDs), radiochromic films, and metal oxide semiconductor field‐effect transistors (MOSFETs).^3^, ^4^, ^5^, ^6^

In external beam radiotherapy, IVTD is employed to monitor the spatial distribution of the radiation dose during treatment. This method involves positioning the patient between the radiation beam and the detector.^7^, ^8^, ^9^, ^10^ In recent years, several authors have published research on IVTD using various detectors, such as ionization chambers,^7^, ^8^ radiochromic films,^9^, ^11^ and electronic portal imaging devices (EPIDs).^11^, ^12^, ^13^, ^14^, ^15^, ^16^, ^17^, ^18^, ^19^ EPIDs are now the most commonly used detectors for IVTD due to their ease of use during patient treatment.^11^, ^12^, ^13^, ^14^, ^15^, ^16^, ^17^, ^18^, ^19^ EPIDs, originally designed to acquire megavoltage portal images during patient treatment to detect positioning errors, also provide dosimetric information. This information can be correlated with the delivered dose, offering the possibility of two‐dimensional^10^, ^12^, ^13^, ^14^, ^15^, ^16^, ^17^, ^18^, ^19^ and three‐dimensional^10^, ^12^, ^20^, ^21^, ^22^ dosimetric data. The most widely used type of EPID is the flat‐panel detector, based on amorphous silicon photodiode (a‐Si) technology, which is now standard on modern linear accelerators (LINACs).^3^, ^10^, ^12^, ^13^, ^14^, ^15^, ^18^ a‐Si EPIDs provide much higher‐quality images and better dosimetric characteristics than previous generations of EPIDs, such as those based on scanning liquid ionization chamber arrays.^10^ Numerous studies have evaluated the performance of on‐line EPID‐based dosimetry and assessed the quality and reproducibility of treatment plans through gamma analysis.

Esposito et al.^18^ demonstrated that EPID‐IVTD can detect treatment parameter errors, such as leaf position, collimator and gantry position, and patient anatomical variations. In these instances, EPID‐IVTD offers a valuable warning for implementing an adaptive strategy. Additionally, the authors suggested that for optimal error detection, on‐line information on tumor and patient position should be integrated with standard pre‐treatment quality assurance (QA) and cone beam computed tomography (CBCT) imaging.^18^

Bossuyt et al.^13^, ^15^ conducted a study on IVTD applied to volumetric modulated arc therapy (VMAT) treatment plans across different anatomical sites, concluding that EPID‐IVTD is valuable for detecting both individual errors and for continuously improving long‐term treatment quality.

In the context of breast radiotherapy, IVTD could be an important tool.^13^, ^14^, ^15^, ^16^, ^17^, ^19^, ^23^ Various sources of error in these treatments may lead to deviations from the planned dose distribution.^15^, ^16^ These errors are primarily due to inaccuracies in patient positioning and changes in breast shape.^19^, ^23^ IVTD allows for careful monitoring of the dose distribution in target areas, which are often close to critical structures such as the ipsilateral lung or heart, thereby minimizing the risk of damage to surrounding healthy tissue.^17^

In a retrospective study by Fiagan et al.,^16^ conducted on more than 200 breast cancer patients, IVTD was used to compare two different treatment prescriptions and assess the dosimetric impact of patient positioning errors and anatomical changes.

Kang et al.^17^ evaluated the robustness of the patient setup procedure for breast treatments in their clinical routine. Specifically, they found that the percentage of low GPRs was three times higher when CBCT was not used before treatment. Their findings highlight that EPID‐IVTD complements the use of CBCT and can provide valuable information for optimizing the treatment process.

Sánchez‐Artuñedo et al.^19^ investigated the dosimetric impact of failing fractions and assessed the appropriateness of using a reference image in breast IVTD. They observed a positive correlation between the GPRs of the transit image and planning target volume (PTV) coverage when using the best fraction as a reference. However, they noted that it is impossible to predict in advance which fraction will best match the treatment plan. They suggested that the best reference fraction might be among the first five.^19^

Bossuyt et al.^15^ used one of the first three EPID images as a reference for EPID‐IVTD, while Bojechko et al.^24^ and Feng et al.^25^ considered the planning‐CT (p‐CT) as the reference.

The choice of the reference fraction is not straightforward and plays a critical role. It involves identifying the treatment fraction that exhibits the highest dosimetric agreement with the original treatment plan. Although a substantial body of literature exists on IVD and EPID‐IVTD in general, to the best of our knowledge, no studies have specifically addressed the effectiveness of a method that takes into account the optimal reference fraction. This aspect is essential for ensuring successful EPID‐IVTD.

The aim of this work was to develop a method for performing accurate EPID‐IVTD in clinical practice for whole‐breast intensity modulated radiation therapy (IMRT). The first phase of the study focused on identifying the fraction to be used as a reference. In the second phase, EPID‐IVTD was used to monitor the agreement between the delivered treatment plans and the reference fraction.

Tolerance limits (TL) and action limits (AL), as introduced by AAPM TG 218,^26^ were employed as criteria for selecting the reference fraction and for monitoring the EPID‐IVTD results in terms of GPRs.

## 2 METHODS AND MATERIALS

**FIGURE 1:**
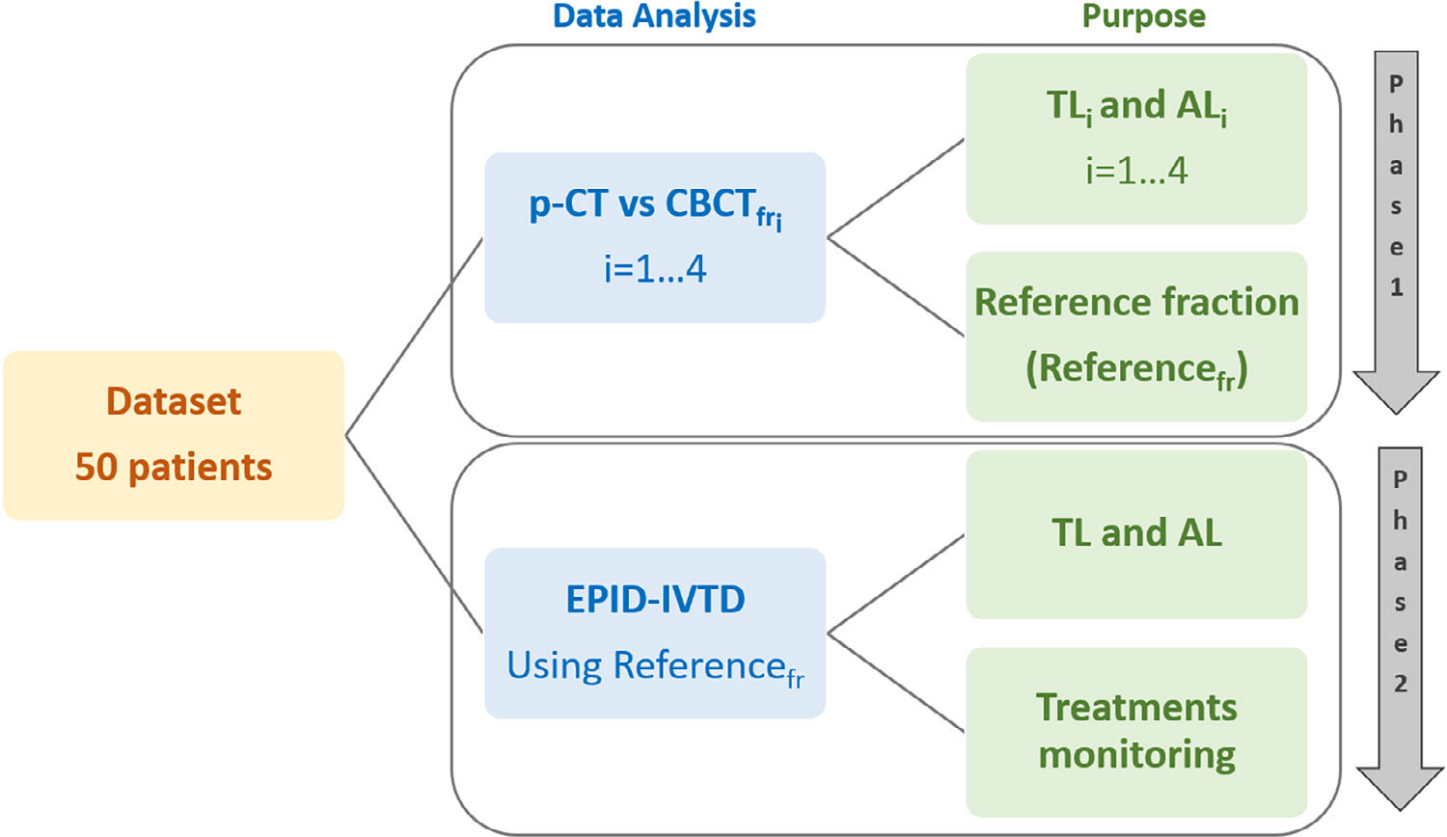
Block diagram illustrating the two phases of the work. The filled boxes in the center describe the two data analysis, while the boxes on the right column indicate the purpose.

A dataset of 50 patients undergoing whole breast IMRT was used for this study. Treatment plans were executed at the Humanitas Istituto Clinico Catanese (HICC), delivered using a Varian TrueBeam 2.7 LINAC, and processed with the Eclipse v. 16.1 Treatment Planning System (TPS) by Varian Medical Systems (Palo Alto, CA). The plans were characterized by hypofractionation with a dose of 2.67 Gy across 15 fractions, two fields (tangential/divergent), fixed jaws, a 6 MV energy beam, and a dose rate of 600 monitor units (MUs) per minute (MU/min). The plans were calculated using the Eclipse Acuros 16.1.2 algorithm with a calculation grid size of 0.15 cm. To account for photon fluence beyond the body contour, the skin flash tool was employed, extending 2 cm beyond the contour. The treatment setup was verified using Align RT (Vision RT) as a surface‐guided radiation therapy (SGRT) system and daily CBCTs. The same dataset of 50 patients was used in both phases of the study (see Figure 1). The objective of the first phase was to identify the fraction with the best dosimetric agreement with the treatment plan, designated as the reference fraction (Reference_fr_). Daily CBCTs acquired during the first four treatment fractions (CBCT_fr1‐4_) were used to perform a dosimetric comparison with the original treatment plan. Planning CTs (p‐CTs) and CBCTs (co‐registered online before treatment) were processed using the TPS contouring module. On the CBCTs, the ipsilateral lung and body structures were contoured. To mitigate Hounsfield unit (HU) discrepancies, HU values were assigned as follows: −700 HU for the lung structure and 0 HU for the body structure on both the p‐CT and CBCTs.^27^, ^28^, ^29^ For each patient, treatment plans were recalculated on the p‐CT (theoretical plan) and on the four CBCTs, using the same MUs as the original plan. The fields were repositioned on the CBCT of each fraction, where the registration displacements, determined immediately before treatment, had been applied. The EPIQA software was then used to perform the dosimetric comparison between the p‐CT and the recalculated treatment fractions on the CBCTs, expressed in terms of global gamma passing rate (GPR) (3%/5 mm, 10% dose threshold). The 3%/5 mm criterion was selected to ensure that the GPR values are comparable, in terms of numerical values, to those typically obtained in our pre‐treatment verifications.

The dosimetric comparisons between the p‐CT and the first four CBCTs, yielded four GPR datasets (p‐CT vs. CBCT_fr1–4_). GPR values were used to compute AL and TL for each dataset. The four GPR datasets were then compared with their respective AL and TL, and the Reference_fr_ was identified by evaluating the GPR values against these limits.

After identifying the Reference_fr_, the second phase involved performing EPID‐IVTD. This was conducted for each fraction of every patient, with the EPID positioned 60 cm from the isocenter to acquire integrated images of the two treatment fields. EPID‐IVTD was executed by comparing the EPID images of all subsequent fractions with the EPID image of the Reference_fr_, which was identified during the first phase.

The EPID used (Varian Medical System a‐Si 1200 model) had an active area of 40 × 40 cm^2^, consisting of a 1190 × 1190 pixel array with a pixel spacing of 0.336 mm.^30^ Gamma analysis was performed using the Varian Portal Dosimetry (PD) software application (v. 2.22.5.0), utilizing the 3%/5 mm and 10% dose threshold criteria. An in‐house custom plug‐in script was developed using the PD scripting application programming interface (Varian Medical Systems, Palo Alto, CA).^31^ This script automatically calculated the GPR by comparing each image with the reference image. The plug‐in was integrated into the PD user interface, allowing access to the currently open patient data. The results, including patient and treatment details, were automatically recorded in a.csv file to facilitate future analysis.

New AL and TL values were derived from GPR data to monitor the EPID‐IVTD performance. Both in the first and the second phase, GPR values greater than 90% were used to calculate ALs and TLs in accordance with AAPM TG N 218^26^. Upper‐ and lower‐AL (u‐AL and l‐AL) were calculated according to Equation (1) ^26^:

(1) $$\Delta A=\beta \;\sqrt{\sigma^{2}+{\left(\bar{x}-T\right)}^{2}}\;$$

where ΔA is the difference between u‐AL and l‐AL. u‐AL was set at 100%, T is the process target value (100%), $\sigma^{2}$ and $\bar{x}$ are respectively the variance and the mean of all the GPRs obtained from group 2. β was set to 6, according to Sanghangthum et al.^27^ Upper‐TL (u‐TL) was set to 100%, instead the lower‐TL (l‐TL) was estimated using the following equation^26^:

(2) $$l-TL=center\;line-2.66\;\cdot \;\bar{mR}$$

where $center\;line=\frac{1}{n}\;\sum_{1}^{n}x$, where n is the total number of GPRs considered and x are the single GPR value. The moving range $\bar{mR}$ is equal to $\frac{1}{n-1}\;\sum_{i=2}^{n}\left|x_{i}+x_{i-1}\right|$.

## 3 RESULT

### 3.1 Reference fraction identification

**FIGURE 2:**
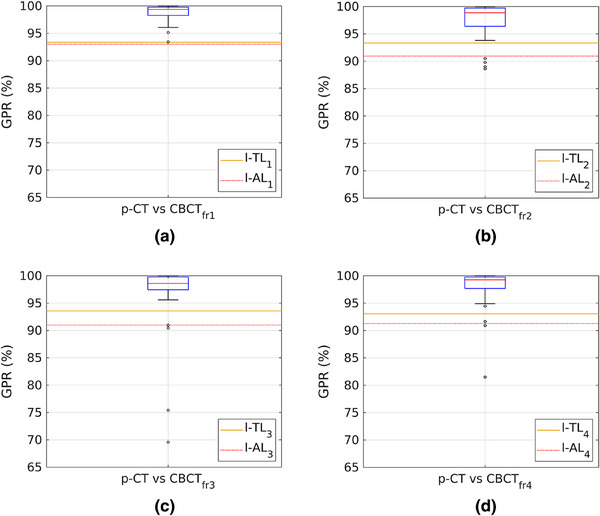
Each graph displays the values of l‐TL (orange line) and l‐AL (red dotted line) obtained from the p‐CT vs. CBCT comparison, along with the box plot of GPRs. From (a) to (d), the data for comparisons from p‐CT vs. CBCT_fr1_ to p‐CT vs. CBCT_fr4_ are shown. CBCT, cone beam computed tomography; GPRs, gamma passing rates; l‐AL, lower‐action limit; l‐TL, lower‐tolerance limit; p‐CT, planning‐CT.

Gamma analysis performed using EPIQA yielded the GPR values displayed in Figure 2, and the TL and AL values reported in Table 1. u‐TL and u‐AL were set at 100%. The results are divided in four datasets based on the comparison between the plans calculated on the p‐CT and those calculated on the CBCTs (p‐CT vs. CBCT_fr1‐4_). The data indicate that the plan recalculated on CBCT_fr1_ exhibits the highest agreement with the theoretical plan. Consequently, the first fraction was selected as the reference for subsequent analyses.

**TABLE 1:** Upper‐ and lower‐TL (u‐TL, l‐TL) and AL (u‐AL, l‐AL) values derived from GPRs analysis conducted for each dataset.

	TL (%)		AL (%)	
	l‐TL	u‐TL	l‐AL	u‐AL
p‐CT vs. CBCT_fr1_	93.37	100	93.00	100
p‐CT vs. CBCT_fr2_	93.34	100	90.94	100
p‐CT vs. CBCT_fr3_	93.58	100	90.99	100
p‐CT vs. CBCT_fr4_	93.05	100	91.28	100

Abbreviations: CBCT, cone beam computed tomography; l‐AL, lower‐action limits; l‐TL, lower‐tolerance limit; p‐CT, planning‐CT; u‐AL, upper‐action limit; u‐TL, upper‐tolerance limit.

### 3.2 EPID‐IVTD results

**FIGURE 3:**
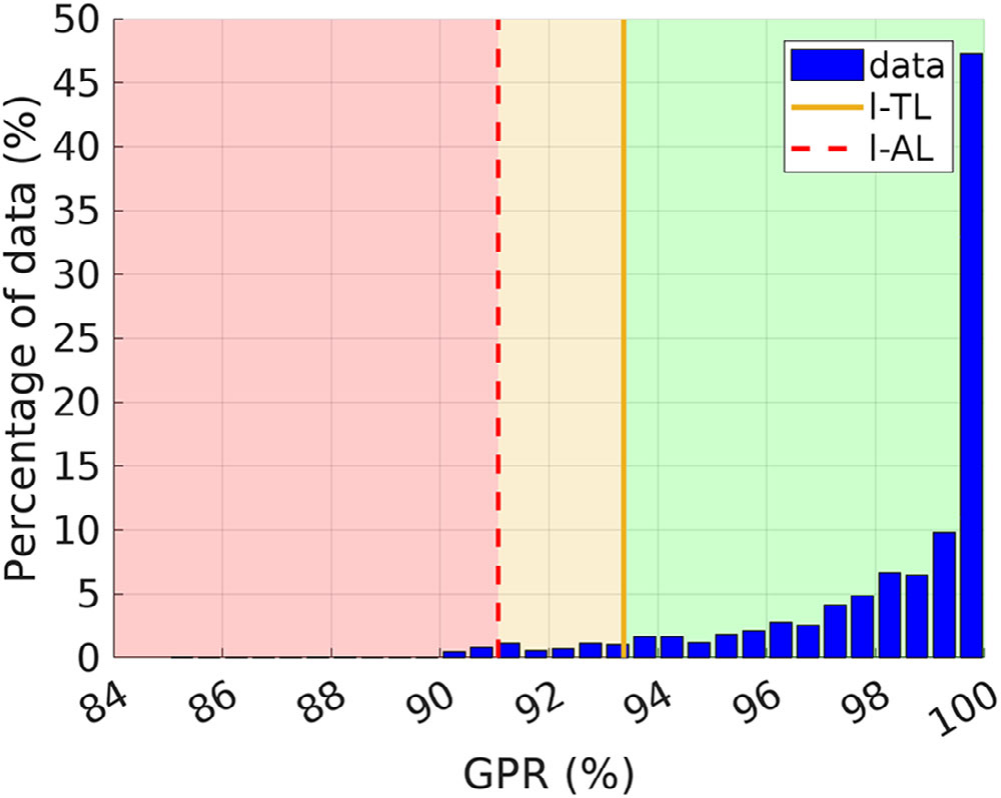
GPR values obtained from EPID‐IVTD, using the first fraction as the reference. l‐TL and l‐AL are represented by the orange line and the red dotted line, respectively. The red area includes GPR values lower than the l‐AL; the orange area includes GPR values between l‐AL and l‐TL; while the green area includes GPR values greater than the l‐TL. EPID, electronic portal imaging device; GPR, gamma passing rate; IVTD, in vivo transit dosimetry; l‐AL, lower‐action limits; l‐TL, lower‐tolerance.

**FIGURE 4:**
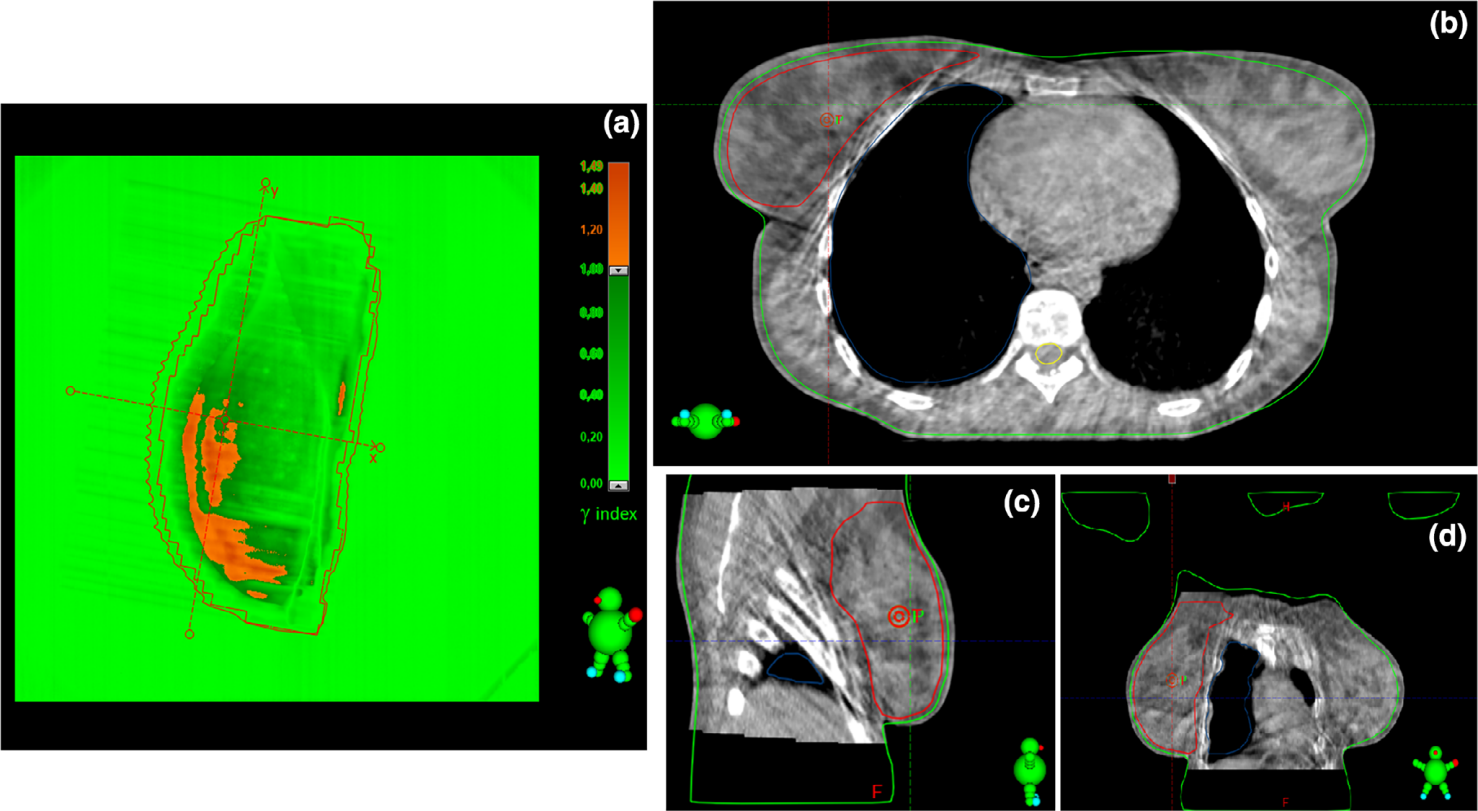
Comparison between the EPID image of the eleventh fraction and the reference fraction using PD (a), offline review of the CBCTfr_11_ vs. p‐CT comparison in the axial (b), sagittal (c), and coronal (d) planes. CBCT, cone beam computed tomography; EPID, electronic portal imaging device; PD, Portal Dosimetry; p‐CT, planning‐CT.

Figure 3 presents the results of the EPID‐IVTD analysis, represented by a histogram of the GPR values obtained from the gamma analysis using the first fraction as the reference. In addition, Figure 3 includes the new l‐TL and l‐AL, calculated to be 93.37% and 91.06%, respectively, while u‐TL and u‐AL remained fixed at 100%. The maximum, minimum, mean, and median GPR values were 100%, 85.3%, 98.25%, and 99.3%, respectively. Additionally, 93.83% of GPR values exceeded the l‐TL, 4.31% fell between the l‐TL and l‐AL, and 1.86% were below the l‐AL threshold. In this phase, the l‐TL and l‐AL values were used as warning and action thresholds, respectively. GPRs exceeding the l‐TL (green area in Figure 3) were considered satisfactory, indicating that EPID‐IVTD was proceeding as expected. GPRs falling between l‐TL and l‐AL (orange area in Figure 3) were noted for caution, while GPR values below l‐AL (red area in Figure 3) required intervention to investigate potential causes. As an example, Figure 4 shows a case of EPID‐IVTD in which the GPR value was below the l‐AL. Figure 4a shows the result of the analysis performed with PD: specifically, the comparison between the eleventh fraction and the Reference_fr_ yielded a GPR value of 90.5%. The comparison between the CBCT acquired on the same day and the p‐CT (Figures 4b–d) revealed a variation in the patient's anatomy. Consequently, a new p‐CT was performed, and the treatment plan was revised.

## 4 DISCUSSIONS

The results from the first phase of this study demonstrated that the best dosimetric agreement between the theoretical plan and the recalculated plan on the first four CBCTs occurred in the p‐CT versus CBCT_fr1_ comparison. This finding aligns with existing literature, where many authors also use the first three fractions^13^, ^15^ or the p‐CT as the reference.^24^, ^25^ Additionally, Sánchez‐Artuñedo et al. suggested that the optimal reference fraction might be found among the first five.^19^ In practice, the EPID‐IVTD methodology proposed in this study relies on the first delivered fraction being in good dosimetric agreement with the theoretical plan, and that this agreement remains consistent throughout subsequent fractions. Achieving this goal may be supported by initiating patient training as early as the p‐CT acquisition phase. Studies have shown that effective communication and patient education by healthcare professionals significantly enhance patient cooperation during radiotherapy.^32^, ^33^ Patient understanding of the technical aspects of treatment and the importance of consistent positioning can greatly improve treatment accuracy.

During the second phase of the study, whenever EPID‐IVTD results (GPRs) fell below l‐TL or l‐AL, the causes were thoroughly investigated. Variations in patient anatomy between fractions and differences in positioning relative to the reference fraction were identified as potential contributors to low GPR values. These factors were identified by comparing EPID images with the reference image and offline daily CBCTs with the p‐CT. Daily CBCT imaging plays a crucial role in identifying these discrepancies, allowing timely adjustments to ensure accurate treatment delivery. Moreover, the use of CBCT has been shown to improve GPR outcomes, as well as enhance dose distribution to the PTV and reduce exposure to organs at risk.^17^ This method of verification also enables decisions regarding the need for re‐planning treatment on a new p‐CT. Additionally, the time interval between the acquisition of the p‐CT and CBCT_fr1_ can impact gamma analysis results. Shortening this interval may reduce the likelihood of significant anatomical changes in the patient, thereby improving treatment accuracy.

Performing gamma analysis for every patient and across all 15 fractions makes EPID‐IVTD a time‐consuming process, particularly when investigating potential causes of failures. However, the implementation of a custom script (see Section 2) successfully reduced the analysis time.

While EPID‐IVTD, as conducted in this study, offers a cost‐effective approach due to the use of standard EPIDs in modern LINAC, a limitation is its inability to provide real‐time feedback. Any errors undetected by CBCT are only identified after the treatment fraction is completed, preventing immediate correction of deviations from the planned dose distribution. This limitation could be addressed by integrating real‐time dose monitoring technologies.^34^, ^35^, ^36^, ^37^, ^38^

## 5 CONCLUSIONS

This study aimed to develop a method for accurately implementing EPID‐IVTD in clinical practice for whole breast IMRT treatments. The results from the first phase suggest that using the first fraction as a reference for EPID‐IVTD is an effective strategy. Based on this finding, the second phase of the study applied our EPID‐IVTD method to the patient cohort. Alert and alarm thresholds were established using l‐TL and l‐AL, respectively. The analysis revealed that 93.83% of GPR values exceeded the l‐TL, 4.31% were between l‐TL and l‐AL, and 1.86% fell below l‐AL. Instances where GPR values fell below l‐TL and/or l‐AL were investigated, and variations in patient anatomy or incorrect positioning were identified as potential factors contributing to low GPR values.

The method developed in this study is now implemented daily at our center, HICC, enabling routine monitoring of breast IMRT treatments.

Future perspectives include implementing a structured breast patient training program and assessing its impact on gamma analysis results. Additionally, extending this research to other breast treatment planning techniques and different anatomical regions could provide further insights into the benefits and limitations of EPID‐IVTD in a broader clinical context.

## AUTHOR CONTRIBUTIONS

*Conceptualization*: Lucia Zirone, Andrea Girlando, Carmelo Marino, and Martina Pace. *Methodology*: Lucia Zirone, Giuseppe Stella, Carmelo Marino, and Martina Pace. *Software*: Lucia Zirone, Giuseppe Stella, Alessia D'Anna, and Martina Pace. *Validation*: Lucia Zirone, Andrea Girlando, Nina Cavalli, Giuseppina Rita Borzì, Elisa Bonanno, Carmelo Marino, and Martina Pace. *Formal analysis*: Lucia Zirone, Giuseppe Stella, Carmelo Marino, and Martina Pace. *Data curation*: Lucia Zirone, Nina Cavalli, Giuseppina Rita Borzì, Giuseppe Stella, Alessia D'Anna, Elisa Bonanno, Carmelo Marino, and Martina Pace. *Investigation*: Lucia Zirone, Carmelo Marino, and Martina Pace. *Writing – Original Draft*: Martina Pace, Lucia Zirone, and Carmelo Marino. *Writing – Review and Editing*: Martina Pace, Lucia Zirone, Carmelo Marino, Giuseppe Stella, and Alessia D'Anna. *Visualization*: Lucia Zirone and Martina Pace. *Supervision*: Lucia Zirone, Giuseppe Stella, Carmelo Marino, and Martina Pace.

## CONFLICT OF INTEREST STATEMENT

The authors declare no conflicts of interest.
